# Protective Effects of Liu Wei Di Huang Wan on the Liver, Orbitofrontal Cortex Nissl Bodies, and Neurites in MSG+PH-Induced Liver Regeneration Rat Model

**DOI:** 10.1155/2018/9090128

**Published:** 2018-08-27

**Authors:** Bin-Bin Zhao, Qing-hua Long, Chao-yang Wang, Lin-lin Chen, Guang-jing Xie, Wen-ji Bo, Bo Xu, Ze-fei Li, Han-Min Li, Ping Wang

**Affiliations:** ^1^Hubei University of Traditional Chinese Medicine, Wuhan 430065, Hubei Province, China; ^2^Hubei Provincial Hospital of Traditional Chinese Medicine (Affiliated Hospital to Hubei University of Chinese Medicine, Hubei Institute of Traditional Chinese Medicine), Wuhan 430065, Hubei Province, China

## Abstract

*Introduction. *To examine the protective effects of Liu Wei Di Huang Wan formula (LWDH) on liver and orbitofrontal cortex (OFC) injuries in monosodium glutamate (MSG) and partial hepatectomy (PH) rat model.* Methods*. Neonatal Wistar rats were given MSG or saline on postnatal days 2, 4, 6, 8, and 10. The rats were caged into five groups and treated accordingly at six weeks old as follows: Saline group, Saline+PH group, MSG group, MSG+PH group, and LWDH group (MSG+PH+LWDH). The PH was performed during week 8 by excision of the left and median hepatic lobes (occupying about 68% of whole liver).On day 8 after the PH, the rats were subjected to an inner OFT before being sacrificed. The liver and OFC were stained using H&E, ORO, or Nissl staining. The expression of neurotrophic factors (*β*-NGF, BDNF) was examined in the OFC lysates by ELISA. Serum levels of cytokines (IL-1*β*, VEGF) were examined using the Bio-Plex suspension array.* Results*. LWDH increased the total distance traveled by the animals (*p*<0.05), and LWDH improved the integrity of the Nissl bodies in the OFC (mean area of the Nissl bodies,* p*<0.01; mean diameter,* p*<0.05; mean density,* p*<0.05; and IOD,* p*<0.01). There were less white area in the liver (*p*>0.05) and decreased hepatic steatosis (*p*<0.01) in LWDH group. LWDH administration decreased the expression of serum levels of IL-1*β* (*p*>0.05), while it increased VEGF (*p*>0.05) expression. LWDH administration increased the expression of BDNF (*p*>0.05) and *β*-NGF (*p*>0.05) in the OFC, all as compared to the MSG+PH group.* Conclusion*. LWDH partly protected the animals from depressive-like behaviors in the MSG+PH-induced liver regeneration neonatal rat model. LWDH alleviated hepatic injury and steatosis and, furthermore, protected the Nissl body integrity and the growth of neurites.

## 1. Introduction

Depression is common in patients with liver disease. There is a higher incidence of depression in patients with chronic liver disease (CLD) when compared to the general population. Data from the 2001-2002 National Epidemiologic Survey on Alcohol and Related Conditions (NESARC), which involves a survey of 43,093 adults aged 18 years and older in the United States, reported an estimated prevalence of liver disease at 0.7%, among which 17.2% reported 12-month rates of major depression, which was significantly higher than the participants without liver disease (7.0%; adjusted OR: 2.2; CI: 1.2-4.1) [1]. Furthermore, participants with comorbid major depression and liver disease showed higher rates of lifetime suicide attempts (33.2%), when compared to the general population (13.7%; OR: 3.1; CI: 1.3-7.6). Despite alcohol use being linked to major depression, liver disease, and suicidal behavior, the sociodemographic factors cannot fully explain the observed association between liver disease and both major depression and suicide. A number of studies have confirmed that patients with liver disease are often accompanied by changes in mood [1–5]. Reciprocally, it has been testified that emotional stimulation can exacerbate liver damage [6]. A recent meta-analysis of 16 prospective studies involving the general population in the United Kingdom from 1994 to 2008 reported that psychological distress, which includes symptoms of anxiety and depression, can be directly associated with increased liver disease mortality [7].

Psychological factors may lead some patients with CLD to seek out alternative treatments, such as herbs and plant preparations. Currently, there is minimal guidance on the proper use of herbal medicines [4]. There is a direct link between hepatic encephalopathy and several symptoms associated with depression, which represent a broad continuum of neuropsychiatric abnormalities [8]. In addition, depression is more prevalent in orthotopic liver transplantation patients, and it has been shown to adversely affect the clinical outcomes [9]. Huang et al. report that Depression and Chronic Liver Diseases may share some underlying mechanisms [10]. Thus, antidepressants are implicated as a combined treatment of the CLD.

Liver regeneration is a well-known postoperative response after partial hepatectomy (PH), representing a unique capacity of the liver. However, whether PHs result in stress and induces depression remains to be studied. The monosodium glutamate- (MSG-) liver regeneration rat model is a well-characterized animal model to study many metabolic abnormalities, such as lipid metabolism and insulin resistance. After undergoing a PH, the MSG-induced animals exhibited disorders of the neuroendocrine-immune network (NEIN) [11, 12]. The MSG-administered rats were shown to have delayed liver regeneration, implicating the involvement of the NEIN in the regulation of liver function [13–15]. Nevertheless, the effects of liver regeneration on depressive-like behaviors resulting from NEIN disorders remain unclear. Recently, Quines* et al*. reported that MSG-induced depressive-like and anxiogenic-like behaviors in rats involved the serotonergic system, synaptosomal serotonin (5-HT) uptake, and monoamine oxidase (MAO-A and MAO-B) activities on the cerebral cortices [16, 17]. These studies provided an ideal model to monitor the effect of liver regeneration on depressive-like symptoms.

The Chinese herbal formulation, Liu Wei Di Huang Wan (LWDH), has been an important part of healthcare in the Chinese Pharmacopeia for hundreds of years. LWDH plays a beneficial role for liver and brain structures upon injuries. MSG+PH-induced liver regeneration rat model shows both liver injury and depressive-like behaviors, providing a suitable model to examine the effect of LWDH on liver injury and orbitofrontal cortex that are mainly responsible for brain injury in depression and the underlying mechanism(s). In the present study, we examined the therapeutic properties of LWDH on liver histology and the depressive-like behaviors by open field test in the MSG+PH-induced liver regeneration rat model. We also stained the orbitofrontal cortex (OFC) to observe the Nissl bodies and to measure the expression of a variety of cytokines.

## 2. Materials and Methods

### 2.1. Reagents

The LWDH was comprised of extracts from six Chinese herbs, including Radix Rehmanniae Preparata (prepared root of* Rehmannia glutiosa*), Fructus Corni (fruit of* Cornus officinalis*), Cortex Moutan (root bark of* Paeonia suffruticosa*), Rhizoma Dioscoreae (rhizome of* Dioscorea opposita*), Poria (scleorotia of* Poria cocos*), and Rhizoma Alismatis (rhizome of* Alisma plantago-aquatica*), in the proportion of 15:15:10:15:15:15. The raw herbs, obtained from the Huangjiahu Hospital of the Hubei University of Chinese Medicine, were boiled in water and filtered. The stock LWDH concentrate was 0.8925 g/mL. MSG was purchased from Sigma-Aldrich (St. Louis, MO).

### 2.2. Establishment of MSG+PH-Induced Liver Regeneration Rat Model and LWDH Administration

Neonatal male Wistar rats were purchased from the Hubei Experiment Animal Research Center. The experiment protocol was approved by the Animal Experiment Ethics Committee of authors' institute. 24 rats were subcutaneously injected with the MSG solution (in normal saline) at a dosage of 4 mg/g body weight, and 16 rats were injected with the same volume of vehicle (saline) on days 2, 4, 6, 8, and 10 after birth. The pups were weaned on day 21 and caged at six weeks old into five groups. The PH was performed during week 8 by excision of the left and median hepatic lobes (occupying about 68% of whole liver) according to the Solt-Farber method under ether anesthesia [18]. The groups were as follows: (1) eight rats that received saline daily via gastrogavage; (2) eight rats that received saline + PH; (3) eight rats that received an MSG injection +saline; (4) eight rats that received MSG +PH; and (5) eight rats with an MSG injection that received LWDH gastrogavage to the endpoint of experiment (1 mL/100g body weight of the LWDH concentrate; daily) +PH. The rats were maintained in an air-conditioned (temperature 24±1°C; 55 ± 5% relative humidity) animal room with controlled lighting (12 h light, 12 h dark). They were provided with commercial diet and water. On day 8 after the PH, the rats were subjected to an inner open field test (OFT) before being sacrificed. The blood, liver, and OFC tissues were collected, before being snap frozen or fixed in formalin. All animal handling and procedures were approved by the Institute of Animal Care and Use Committee of the Hubei University of Chinese Medicine.

### 2.3. Inner Open Field Test

The inner OFT was conducted in an open arena (l, w, h: 50cm ×50cm ×40 cm), with the bottom and sides, made of Plexiglas covered with black, nonreflecting material, and placed in a quiet room. On day 8 after the PH, the animals were individually placed in the center of the apparatus and were left to move freely during a 3 min period with their movements being automatically recorded using a camera connected to a computer (ZH-ZFT, Zhenghua Biological Instrument Co., Ltd., Huaibei, China). The total distance moved (cm) and total distance traveled (cm) in the center area were analyzed.

### 2.4. Histology and Immunohistochemistry Staining

After dewaxing and hydration of the paraffin sections, the liver and cortex tissues were stained with hematoxylin and eosin (H&E). The liver tissue sections were stained with Oil Red O (ORO). Nissl staining was performed on the OFC sections for Nissl body observation. Five fields from each slide were randomly selected, viewed and imaged under a fluorescence microscope (Nikon TE2000-U, Nikon, Japan), and analyzed using the Image Pro-Plus 6.0 software (Media Cybernetics, Silver Spring, MD). The minimal pixel was set at 50 pixels, and the average and cumulative optical density values, average area, and average diameter were analyzed.

### 2.5. Measurement of Cytokines

Fifty mg frozen OFC was lysed on ice for protein extraction. Using ELISA kits (Ray Biotech, Norcross, GA), *β*-nerve growth factor (NGF sensitivity: 14 pg/ml; CV%: <10%) and brain-derived neurotrophic factor (BDNF sensitivity: 12 pg/ml; CV%: <10%) were measured according to the manufacturer's protocol. After the animals were sacrificed, the serum was removed for analysis. The levels of IL-1*β*, IL-10, vascular endothelial growth factor) VEGF, and tumor necrosis factor- (TNF-) *α* were measured using the Bio-Plex suspension array system (Bio-Rad Laboratories, Berkeley, CA).

### 2.6. Statistical Analyses

Data were expressed as the mean ± standard deviation (SD). Statistical analyses were performed using the SPSS software (version 19.0; IBM, Armonk, NY). Intergroup comparisons were performed using the one-way analysis of variance (ANOVA), and* p*-values of less than 0.05 were considered as statistically significant.

## 3. Results

### 3.1. Depressive-Like Behaviors

**Figure 1:**
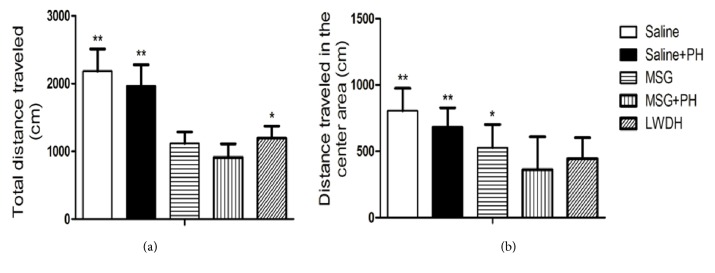
**Effect of LWDH administration on the depressive-like behaviors of neonatal rats. **(a) The total traveled distance of different groups of animals in the open arena; (b) distance traveled in the center area of the open arena. ^*∗*^*p*<0.05, compared to MSG+PH group; ^*∗∗*^*p*<0.01, compared to MSG+PH group. N=8 per group. Saline: NaCl 0.9% subcutaneous/ip+NaCl 0.9% per oral/po in rats; Saline+PH: NaCl 0.9% subcutaneous/ip+NaCl 0.9% per oral/po in rats; MSG: MSG 4000 mg/kg bw (ip)+NaCl 0.9% (po) in rats; MSG+PH: MSG 4000 mg/kg bw (ip)+PH+NaCl 0.9% (po) in rats; LWDH: MSG 4000 mg/kg bw (ip)+PH+LWDH 8925 mg/kg bw (po) in rats.

We first examined the effect of LWDH on the depressive-like behaviors of neonatal rats. As shown in Figure 1, the saline-treated animals without a PH traveled a total distance of 2183.34 ± 327.52 cm in 3 min (Figure 1(a)) and the distance traveled in the center of the open arena was 804.44 ±169.32 cm (Figure 1(b)). The saline-treated animals with PH traveled a total distance of 1964.87 ± 313.72 cm and the distance traveled in the center was 684.73 ± 142.89 cm. The MSG-induced rats without a PH traveled a total distance of 526.85 ± 174.62 cm in the open arena. In contrast, the MSG-induced animals with a PH traveled a significantly shorter total distance (910.11 ± 202.25 cm;* p*<0.01, compared with saline group;* p*<0.05, compared with saline+PH group), as well as the distance traveled in the center of the arena (360.53 ± 248.24 cm;* p*<0.01, compared with saline group;* p*<0.05, compared with saline+PH group and MSG group without PH). These results confirmed the presence of depressive-like behaviors in the established MSG+PH-induced animal model. The administration of LWDH to MSG+PH-induced animals showed significantly increased total traveled distance (1192.86 ±180.41 cm;* p*<0.05), as compared to the MSG+PH-induced animals. However, there was no significant difference in the distance traveled in the center arena (360.53 ± 248.24 cm versus 443.89 ± 158.14 cm). Therefore, LWDH could partially alleviate depressive-like behaviors in the MSG+PH-induced liver regeneration model.

### 3.2. Histological Analysis of Liver

**Figure 2:**
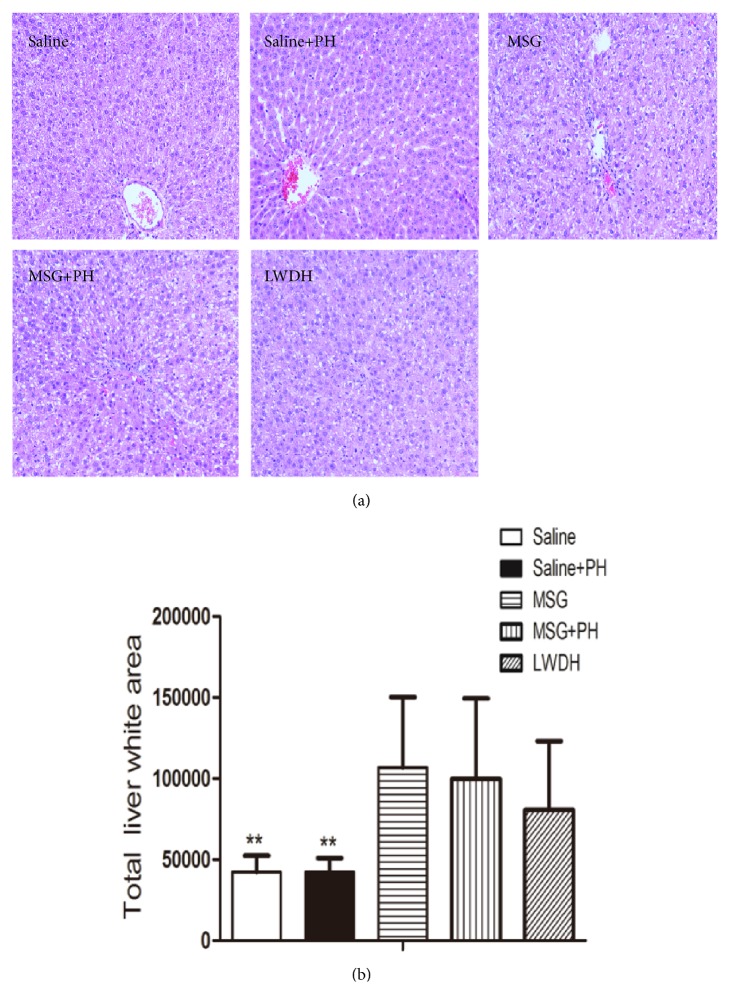
**Effect of LWDH on the liver histology of neonatal rats. **Histological analysis of the liver after partial hepatectomy with LWDH administration. (a) Sections were stained using hematoxylin and eosin. All pictures were taken at 200 magnification (200×). (b) Statistical analysis of the liver white area of different groups of animals. ^*∗∗*^*p*<0.01, compared to MSG+PH group. N=8 per group. Saline: NaCl 0.9% subcutaneous/ip+NaCl 0.9% per oral/po in rats; Saline+PH: NaCl 0.9% subcutaneous/ip+NaCl 0.9% per oral/po in rats; MSG: MSG 4000 mg/kg bw (ip)+NaCl 0.9% (po) in rats; MSG+PH: MSG 4000 mg/kg bw (ip)+PH+NaCl 0.9% (po) in rats; LWDH: MSG 4000 mg/kg bw (ip)+PH+LWDH 8925 mg/kg bw (po) in rats.

Liver histology in saline- and MSG-injected rats was analyzed using H&E staining. The liver sections in animals that received saline or LWDH gastrogavage on day 8 post-PH are illustrated in Figure 2. Normal liver histology with typical lobular architecture was observed in rats of the saline group. After a two-thirds PH, the liver of the neonatal rats was nearly restored to its initial architecture with only a few small fat vacuoles by 8 days after surgery. In contrast, animals in the MSG-induced liver group exhibited diminished borderlines of the liver lobes with loosening cytoplasmic structures of the hepatocytes. There were increased hepatic vacuoles and the liver cells, especially those located centrally in the lobuli, which displayed considerable swelling with vacuolization and a balloon-like appearance. Following the PH, the MSG-induced rats exhibited a higher degree of disturbances of structure in the liver, as evidenced by the disappearance of borderlines and more balloon-like hepatocytes and vacuolization. The administration of LWDH to the MSG+PH-induced animals attenuated the changes in liver architecture. There were slight decreases in balloon-like hepatocytes and vacuolization (Figure 2(a)). We further analyzed the white areas in the H&E stained slides. The unknown tiny particles were discarded by setting a minimum pixel value to 50, and the white portal areas were excluded by setting a maximum pixel value to 5000. As shown in Figure 2(b), the MSG+PH animals had a significantly larger white area in the liver architecture, 99714.85 ± 49745.45, as compared to the saline-treated rats (42226.44 ± 10143.22;* p*<0.01) and saline-treated PH animals (42173.78 ± 8793.32;* p*<0.01).

### 3.3. Analysis of Liver Steatosis

**Figure 3:**
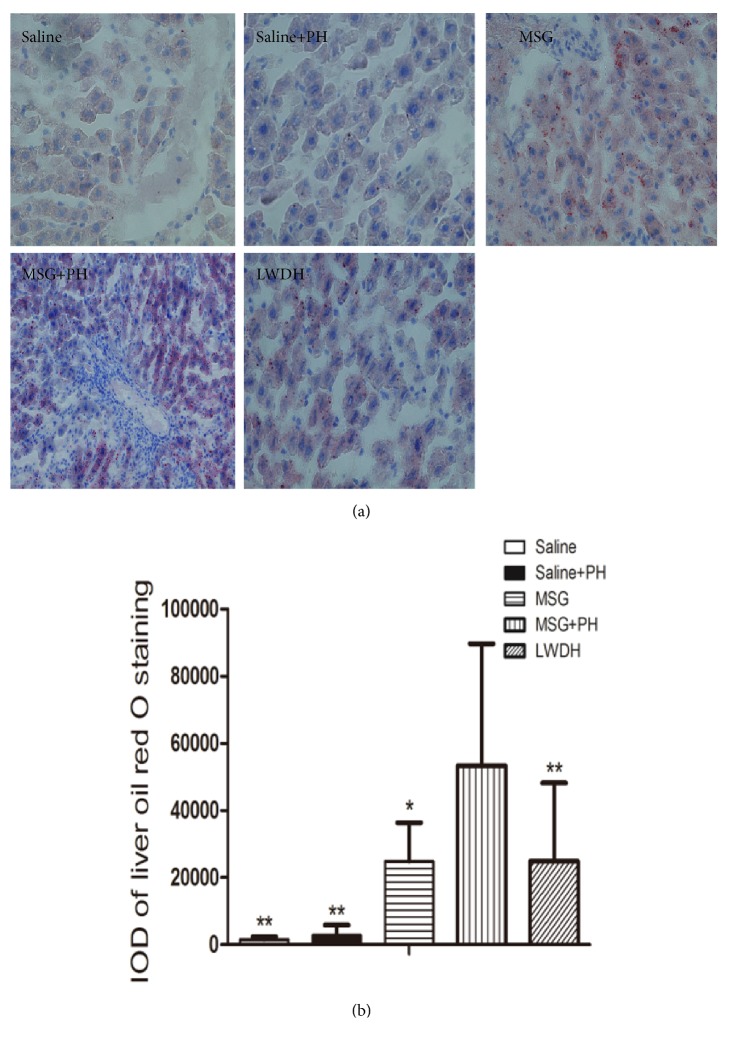
**Effect of LWDH on hepatic steatosis of neonatal rats. **Oil-red O staining of the liver after partial hepatectomy and LWDH administration. Sections of frozen liver tissues were stained with Oil Red O. All pictures were taken at 200 magnification (400×). ^*∗*^*p*<0.05, ^*∗∗*^*p*<0.01, compared to MSG + PH group. N=8 per group. Saline: NaCl 0.9% subcutaneous/ip+NaCl 0.9% per oral/po in rats; Saline+PH: NaCl 0.9% subcutaneous/ip+NaCl 0.9% per oral/po in rats; MSG: MSG 4000 mg/kg bw (ip)+NaCl 0.9% (po) in rats; MSG+PH: MSG 4000 mg/kg bw (ip)+PH+NaCl 0.9% (po) in rats; LWDH: MSG 4000 mg/kg bw (ip)+PH+LWDH 8925 mg/kg bw (po) in rats.

We next sought to analyze the hepatic steatosis. The frozen liver tissues were sectioned and stained with ORO solution. The saline group without the PH had an integral optical density (IOD) of 1550.62 ± 834.14 and the saline+PH group had an IOD of 2803.54 ± 3080.47. The MSG-induced animals without the PH had an IOD of 24872.82 ± 11495.65, which was significantly higher than that of the saline without the PH and saline+PH groups,* p*<0.01. As compared to the MSG-induced rats with the PH operation that exhibited an IOD of 53352.49±36345.17, LWDH administration during the recovery period after PH operation significantly alleviated the liver steatosis (24880.22±23328.13;* p*<0.05; Figure 3). These results demonstrated that MSG-induced rats had significant liver steatosis and the PH operation increased the level of steatosis. However, treatment with LWDH could protect the animals from PH and MSG-induced hepatic steatosis.

### 3.4. Histopathological Analyses of OFC

**Figure 4:**
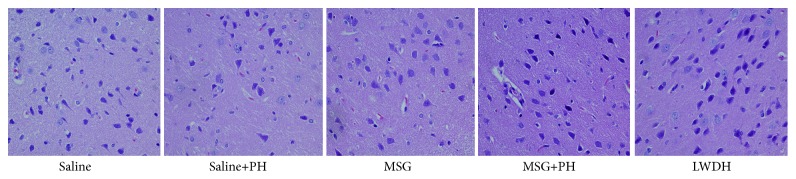
**Effect of LWDH on histology of the orbitofrontal cortex of neonatal rats. **Sections were stained using hematoxylin-eosin. All pictures were taken at 400 magnification (400×). Saline: NaCl 0.9% subcutaneous/ip+NaCl 0.9% per oral/po in rats; Saline+PH: NaCl 0.9% subcutaneous/ip+NaCl 0.9% per oral/po in rats; MSG: MSG 4000 mg/kg bw (ip)+NaCl 0.9% (po) in rats; MSG+PH: MSG 4000 mg/kg bw (ip)+PH+NaCl 0.9% (po) in rats; LWDH: MSG 4000 mg/kg bw (ip)+PH+LWDH 8925 mg/kg bw (po) in rats.

H&E staining of the OFC is shown in Figure 4. The neurons of the saline-treated rats had integral morphology and structure with normal synapses. Saline-treated rats with the PH operation had relatively integral neurons, yet some of the neurons exhibited shrunken soma and reduced synapses. In contrast, the neurons in the OFC of the MSG-induced animals were largely damaged, exhibiting reduced size in the neuronal soma and disappearance of the synapses. These effects were even more apparent in MSG-induced animals after the PH surgery. The morphology of the cortical neurons recovered in the animal administered with LWDH during the postoperative period.

### 3.5. Nissl Body and Neurite Observation

**Figure 5:**
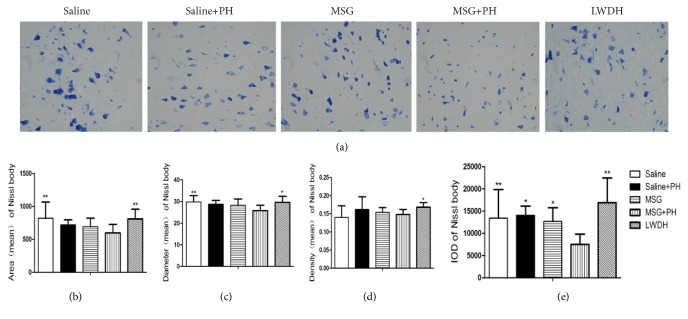
**Effect of LWDH on Nissl body and neurite structure in the orbitofrontal cortex of neonatal rats. **The orbitofrontal cortex of animals with different treatments were sectioned and stained. (a) Representative morphology of the Nissl bodies and neurites of each group of animals are shown. The mean area (b), diameter (c), densitometry (d), and IOD (e) of individual groups were statistically analyzed. N=8 per group. ^*∗*^*p*<0.05; ^*∗∗*^*p*< 0.01, as compared with MSG+PH group. 400 magnification (400×). Saline: NaCl 0.9% subcutaneous/ip+NaCl 0.9% per oral/po in rats; Saline+PH: NaCl 0.9% subcutaneous/ip+NaCl 0.9% per oral/po in rats; MSG: MSG 4000 mg/kg bw (ip)+NaCl 0.9% (po) in rats; MSG+PH: MSG 4000 mg/kg bw (ip)+PH+NaCl 0.9% (po) in rats; LWDH: MSG 4000 mg/kg bw (ip)+PH+LWDH 8925 mg/kg bw (po) in rats.

We next sought to investigate the protective effect of LWDH on the cortical Nissl bodies and neurite growth (Figure 5). As shown in Figure 5(a), the saline-injected animals showed integral Nissl bodies in the OFC with long and thick neurites. The mean area was 820.70±244.85, the mean diameter of the Nissl bodies was 29.81±2.96, and the IOD of the stained tissue was 13436.79±6416.82. The PH operation decreased the volume of the Nissl bodies. The neurons exhibited abnormal morphology, including shortening and thinning of the neurites (Figures 5(a), 5(b), and 5(c)). This was also significant in the frontal cortex of the MSG-induced rats with the disappearance of some parts of the synapses. The PH operation in the MSG-induced rats showed significantly decreased staining of the neurons (Figures 5(a) and 5(e)) and disappearance of almost all synapses, with a mean area of 597.52±128.18, mean diameter of the Nissl bodies of 25.81±2.51, and IOD of 7491.99±2317.19*, p*<0.01, all as compared to the Saline group. In contrast, we observed that administration of LWDH largely protected the integrity of the Nissl bodies and the morphology of the neurites (mean area of 809.87±148.33,* p*<0.01; mean diameter of the Nissl bodies of 29.62±2.78,* p*<0.05; mean density of 0.168±0.013,* p*<0.05; and IOD of 16876.76±5597.77,* p*<0.01; all as compared to the MSG+PH group) as shown in Figures 5(a), 5(b), and 5(e).

### 3.6. Growth Factors and Cytokine Expression

**Figure 6:**
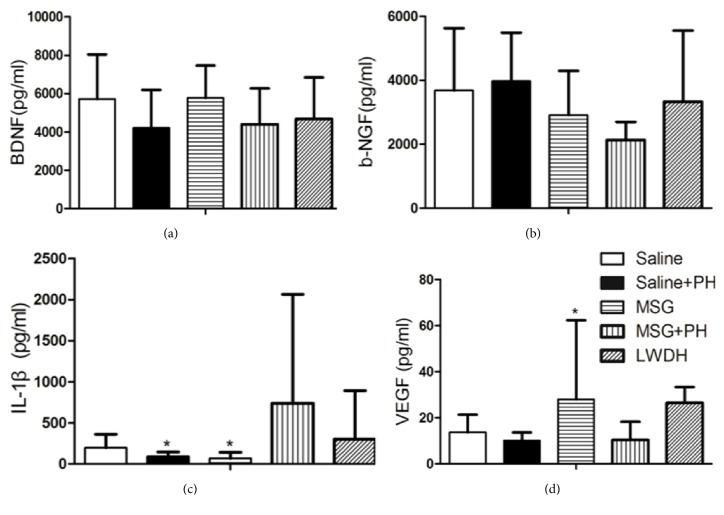
**Effect of LWDH on the expression of neurotrophic factors in orbitofrontal cortex and serum cytokines in the neonatal rats. **(a, b) The expression of BDNF and *β*-NGF in the orbitofrontal cortex. (c, d) The expression of IL-1*β* and VEGF in the serum of different groups. N=8 per group. ^*∗*^*p*<0.05, as compared to MSG+PH group. Saline: NaCl 0.9% subcutaneous/ip+NaCl 0.9% per oral/po in rats; Saline+PH: NaCl 0.9% subcutaneous/ip+NaCl 0.9% per oral/po in rats; MSG: MSG 4000 mg/kg bw (ip)+NaCl 0.9% (po) in rats; MSG+PH: MSG 4000 mg/kg bw (ip)+PH+NaCl 0.9% (po) in rats; LWDH: MSG 4000 mg/kg bw (ip)+PH+LWDH 8925 mg/kg bw (po) in rats.

There were no significant differences in the levels of BDNF and *β*-NGF present in the OFC between the different groups of animals (Figures 6(a) and 6(b)). The serum levels of IL-1*β* (Figure 6(c)) were 736.15±1327.19 pg/mL in MSG-induced rats with the PH operation, significantly upregulated in comparison to the Saline+PH group (91.51 ± 53.37 pg/mL,* p*<0.05) and MSG-induced rats (67.50 ± 73.33 pg/mL,* p*<0.05). However, MSG+PH-induced animals showed decreased serum levels of VEGF (10.39±7.92pg/mL,* p*<0.05), as compared to the MSG treatment alone (27.96 ± 34.43 pg/mL, Figure 6(d)). The administration of LWDH slightly reversed the effects of PH on MSG-treated animals (it slightly decreased IL-1*β*, while it increased VEGF), which might contribute to the protective effect of LWDH in the MSG+PH-induced liver regeneration model.

## 4. Discussion

In the present study, we revealed that MSG+PH-induced liver regeneration model rats displayed impaired locomotive activity with depressive-like behaviors. The PH operation in MSG-induced animals resulted in damage to the Nissl bodies and diminished neurite growth, which also impaired liver regeneration. LWDH, a traditional Chinese herbal formulation, partly protected the animals from depressive-like behaviors in the MSG+PH-induced liver regeneration neonatal rat model. LWDH alleviated liver injury and hepatic steatosis. LWDH protected the Nissl body integrity and the growth of neurites.

The MSG-administered rats were treated during the first ten postnatal days with MSG. The MSG administration induced hypothalamic neurotoxicity accompanied by metabolic disorders, including obesity, a transient insulin resistance, and metabolic alterations, demonstrated in the blood, liver and skeletal muscle, and lipotoxicity, characterized in the liver and skeletal muscle [19]. In an earlier study, we have shown that MSG is a kind of neurotoxin, as its administration to newborn rats could selectively damage the arcuate nucleus portion of the hypothalamus, which could lead to dysfunction of hypothalamus-pituitary-target gland axis and result in the swelling and necrosis in neurocytes. This is believed to be a possible mechanism underlying MSG-associated depressive behaviors [13, 14].

The liver has a unique capacity to regenerate itself after a PH. Here, we showed that MSG+PH-induced rats had disturbed regenerative processes, evidenced by the increased white area and hepatic steatosis. At early time points after surgery (within 24 h), there was rapid liver regeneration. However, the process was significantly suppressed by the later time points after the PH operation. The extent of liver regeneration, division index of hepatocytes, and ratio of the liver weight to body weight was significantly impaired in MSG+PH-induced rats [14]. These results suggest that extra hepatic factors, such as the immunomodulatory impact by the central nervous system (CNS), may influence the process of liver regeneration. The hypothalamus-pituitary-liver axis and the regulatory network for NEIN-associated liver regeneration have been implicated in these processes [20, 21].

Chinese medicine has a long history in the treatment of acute and chronic liver diseases. The mechanisms underlying the protective effect of these traditional Chinese formulations usually involve multilevel and multifactorial regulation of the regenerative process with multiple targets. The NEIN has been implicated in the regulation of liver regeneration [14]. Diverse endocrine and neuroendocrine interactions, as well as the recruitment of blood cells, are known to affect liver regeneration. We previously reported that the formulation, Zuo Gui Wan (ZGW), can regulate the NEIN and consequently modulate liver regeneration [20, 21]. As compared to the liver of normal rats that underwent the PH operation, there was decreased expression of TGF-*α* (at both mRNA and protein levels) and EGFR in the regenerated liver tissues. However, the expression of TGF-*β*1 and TGF-*β* type I receptors (TGFBRI and TGFBRII) were upregulated in the ARN of MSG-induced rats. In contrast, ZGW could significantly increase the expression of TGF-*α* and EGFR, while decreasing the expression of TGF-*β*1 and TGF-*β* receptors. Another Chinese herbal formulation, known as Di Wu Yang Gan (DWYG), has been used in the prevention and treatment of liver injuries. It has also been shown to increase 2-acetylaminofluorene (2-AAF) and the survival rates of rats undergoing the PH procedure. Additionally, DWYG suppresses hepatic precarcinoma changes and restores hepatic tissue structure and functions [18]. DWYG might ameliorate the tissue regenerative environment of the liver via the hypothalamus-pituitary-liver axis [22]. These earlier studies laid a solid foundation for our current investigation by establishing a suitable animal model of liver regeneration with NEIN disorders. Liver injuries and hepatic steatosis were evidenced in the MSG+PH-induced rats. The LWDH could prevent and treat structural abnormalities and steatosis of the liver, demonstrating the protective effect of the formulation. We observed increased serum levels of IL-1*β* in MSG+PH-induced rats as compared with MSG-induced alone, suggesting that the PH activated liver regeneration. During the inflammatory phase after parenchymal injury of the liver, the activation of Kupffer cells, mononuclear phagocytes, lymphocytes, and platelets elicited the secretion of a variety of inflammatory cytokines. These cytokines, including IL-1*β*, IL-6, TNF-*α*, and TGF-*β*, are believed to have a role in the paracrine regulation of adipocyte proliferation and fibrosis.

The OFC has been implicated in the pathophysiology of major depression by evidence obtained through neuroimaging, neuropathology, and lesion analysis techniques [15]. Increased functional connectivity of the lateral OFC Brodmann area 47/12 is related to depression [23].The neuronal soma was diminished in the OFC of MSG+PH-induced rats, and the synapses were decreased in comparison to the MSG groups. The administration of LWDH to the animals largely protected the integrity of the Nissl bodies and neurites, demonstrating that LWDH contributes to the increased neuroplasticity. Neurotrophic factors (NTFs) are a family of growth factors that support the growth, survival, and differentiation of both developing and mature neurons. NTFs play pivotal roles in the formation and plasticity of neuronal networks in adult brains [21]. Among the NTFs, BDNF is a major neuronal growth factor in the brain, which regulates neurogenesis, neuronal maturation and survival, and synaptic plasticity. The deficiency of BDNF in the prefrontal cortex and hippocampus is known to impair brain function, which can result in depressive disorders. Locally synthesized BDNF in the dendrites of granule cells has been shown to promote differentiation and maturation of progenitor cells in the subgranular zone (SGZ) by enhancing gamma-aminobutyric acid (GABA) release [24]. BDNF is believed to influence the activity of the serotonergic, noradrenergic, and dopaminergic pathways, and reduced BDNF concentration is implicated in poor sleep quality [25]. As such, elevated levels of BDNF in the regions within the orbital and medial prefrontal cortices, which can integrate sensory information and increase the outgrowth of neurites and the neuroplasticity, may decrease depression. Indeed, treadmill exercise after social isolation has been reported to increase the levels of NGF, BDNF, and synapsin I, which induces the survival of neurons in the hippocampus and effectively eliminates the depressive-like behaviors [26]. NGF is another neuroregulatory molecule that can provide nutrients for neurons and promote the outgrowth of neurites. NGF is involved in the pathophysiology of major depression disorder (MDD) and suicide risk (SR), and reduced NGF has been recommended as a serum biomarker for MDD [27]. Because serum levels of BDNF and *β*-NGF are less relevant to brain injuries, we next measured the expression of BDNF and *β*-NGF in the OFC tissue lysates. We observed a slight decrease in these two NTFs in the MSG+PH-induced animals and LWDH could attenuate the decrease.

In addition, the administration of LWDH increased the serum levels of VEGF, a growth factor known to promote vascular growth and maintain the vascular integrity. VEGF is secreted by a variety of cells, including vascular endothelial cells and several types of brain cells (astrocytes, ependymal cells, and neural stem cells). VEGF has been implicated as neurotrophic and neuroprotective in the peripheral nervous system (PNS) and CNS, directly influencing Schwann cells, neuronal progenitor cells, astrocytes, and microglia [28]. However, the blood VEGF levels in patients with MDD were significantly higher than those in the healthy controls, and the difference was negatively correlated with the mean age [29].VEGF and VEGFR2 increased significantly in the prefrontal cortex after chronic restraint stress, and there was a tendency towards increased serum VEGF protein expression after both acute and chronic restraint stress [30]. Experiments in animal models of depression have also demonstrated that VEGFR2 signaling is indispensable for cellular and behavioral response to antidepressant drugs [28]. Therefore, the regulation of VEGF by LWDH may underlie the protective effect of LWDH on the depressive-like behaviors in the MSG+PH-induced liver regeneration model.

In summary, our study revealed that LWDH could significantly promote liver regeneration and alleviate hepatic steatosis in the MSG+PH-induced liver regeneration model. In addition, LWDH partly protected the animals from depressive-like behaviors and neuronal injuries.

## Data Availability

The data used to support the findings of this study are included within the article.
